# Effects of Exercise on Heart Failure with Preserved Ejection Fraction: An Updated Review of Literature

**DOI:** 10.3390/jcdd9080241

**Published:** 2022-07-28

**Authors:** Giulia Crisci, Mariarosaria De Luca, Roberta D’Assante, Brigida Ranieri, Anna D’Agostino, Valeria Valente, Federica Giardino, Valentina Capone, Salvatore Chianese, Salvatore Rega, Rosangela Cocchia, Muhammad Zubair Israr, Radek Debiek, Liam M. Heaney, Andrea Salzano

**Affiliations:** 1 Department of Translational Medical Sciences, Federico II University, 80131 Naples, Italy; criscigiulia21@gmail.com (G.C.); deluca.mrs@libero.it (M.D.L.); roberta.dassante@outlook.it (R.D.); valeriaelettravalente@gmail.com (V.V.); federicagiardino94@gmail.com (F.G.); salreg25@gmail.com (S.R.); 2 Italian Clinical Outcome Research and Reporting Program (I-CORRP), 80131 Naples, Italy; 3 IRCCS Synlab SDN, Diagnostic and Nuclear Research Institute, 80143 Naples, Italy; ranieribrigida@gmail.com (B.R.);; 4 Division of Cardiology, AORN Antonio Cardarelli Hospital, 80131 Naples, Italy; caponevalentina92@libero.it (V.C.); sasichian@gmail.com (S.C.); rosangelacocchia@hotmail.com (R.C.); 5 Department of Cardiovascular Sciences, University of Leicester and NIHR Leicester Biomedical Research Centre, Leicester LE5 4PW, UK; mzi4@leicester.ac.uk (M.Z.I.); rmd24@leicester.ac.uk (R.D.); 6 School of Sport, Exercise and Health Sciences, Loughborough University, Loughborough LE11 3TU, UK; l.m.heaney2@lboro.ac.uk

**Keywords:** exercise intolerance, heart failure, exercise, heart failure with preserved ejection fraction, cardiopulmonary exercise test, peak VO_2_

## Abstract

Heart failure with preserved ejection fraction (HFpEF) represents the most common HF phenotype of patients aged > 65 years, with an incidence and a prevalence that are constantly growing. The HFpEF cardinal symptom is exercise intolerance (EI), defined as the impaired ability to perform physical activity and to reach the predicted age-related level of exercise duration in the absence of symptoms—such as fatigue or dyspnea—and is associated with a poor quality of life, a higher number of hospitalizations, and poor outcomes. The evidence of the protective effect between exercise and adverse cardiovascular outcomes is numerous and long-established. Regular exercise is known to reduce cardiovascular events and overall mortality both in apparently healthy individuals and in patients with established cardiovascular disease, representing a cornerstone in the prevention and treatment of many cardio-metabolic conditions. Several studies have investigated the role of exercise in HFpEF patients. The present review aims to dwell upon the effects of exercise on HFpEF. For this purpose, the relevant data from a literature search (PubMed, EMBASE, and Medline) were reviewed. The analysis of these studies underlines the fact that exercise training programs improve the cardiorespiratory performance of HFpEF patients in terms of the increase in peak oxygen uptake, the 6 min walk test distance, and the ventilatory threshold; on the other hand, diastolic or systolic functions are generally unchanged or only partially modified by exercise, suggesting that multiple mechanisms contribute to the improvement of exercise tolerance in HFpEF patients. In conclusion, considering that exercise training programs are able to improve the cardiorespiratory performance of HFpEF patients, the prescription of exercise training programs should be encouraged in stable HFpEF patients, and further research is needed to better elucidate the pathophysiological mechanisms underpinning the beneficial effects described.

## 1. Introduction

Heart failure with preserved ejection fraction (HFpEF) is defined as a clinical syndrome characterized by typical symptoms (e.g., breathlessness and fatigue) and signs (e.g., peripheral oedema and lung crackles), evidence of cardiac structural and/or functional abnormalities—consistent with the presence of left ventricle diastolic dysfunction/raised left ventricle filling pressures—and left ventricular ejection fraction (LVEF) ≥ 50% [1,2].

Notably, HFpEF represents the most common HF phenotype of patients aged > 65 years [3], with a constantly growing incidence and prevalence [4] due to the ageing of the general population and the increasing prevalence of conditions associated with HFpEF development (i.e., obesity, metabolic syndrome, and diabetes mellitus) [5,6,7].

Nowadays, HFpEF represents a major cause of morbidity and mortality. It is a complex syndrome characterized by multi-organ involvement; it is typical in older, female patients and is frequently correlated with obesity, hypertension, ischemic heart disease, diabetes mellitus, and atrial fibrillation [8,9,10,11], and it may present with a wide range of clinical pictures, ranging from asymptomatic or mild disease to life-threatening conditions [1].

The diagnosis of HFpEF is challenging, and in recent years, functional testing (i.e., to detect hemodynamic abnormalities during exercise) has been clearly proven as important in HFpEF diagnosis [12]. Indeed, there is an intimate link between the (expected) hemodynamical changes during exercise and the abnormalities described in HFpEF, with some not present at rest and detectable only during exercise [12]

Finally, exercise intolerance (i.e., the impaired ability to perform physical activity and to reach the predicted age-related level of exercise duration in the presence of symptoms—such as fatigue or dyspnea) is a typical feature of HFpEF and is associated with a poor quality of life, a higher incidence of hospitalization, and poor outcomes [13].

Taking these premises into account and shedding light upon the intimate link between exercise and HFpEF, the present review aims to dwell upon the effects of exercise on HFpEF. For this purpose, the relevant data from a literature search (PubMed, EMBASE, and Medline) were reviewed. After a brief introductive paragraph on the general benefits of exercise on cardiovascular performance, with an excursus on the role of exercise on primary and secondary prevention, the available evidence regarding the effect of exercise in HFpEF is revised; finally, the future perspectives with regard to the role of exercise in the management and treatment of HFpEF are discussed.

## 2. Cardiovascular Benefits of Exercise

The evidence of an inverse relationship between exercise and adverse cardiovascular outcomes is numerous and long-established [14,15]. Indeed, regular exercise is known to reduce cardiovascular events and overall mortality in both apparently healthy individuals and patients with established cardiovascular disease, representing a cornerstone in the prevention and treatment of many cardio-metabolic conditions [16,17].

**Figure 1:**
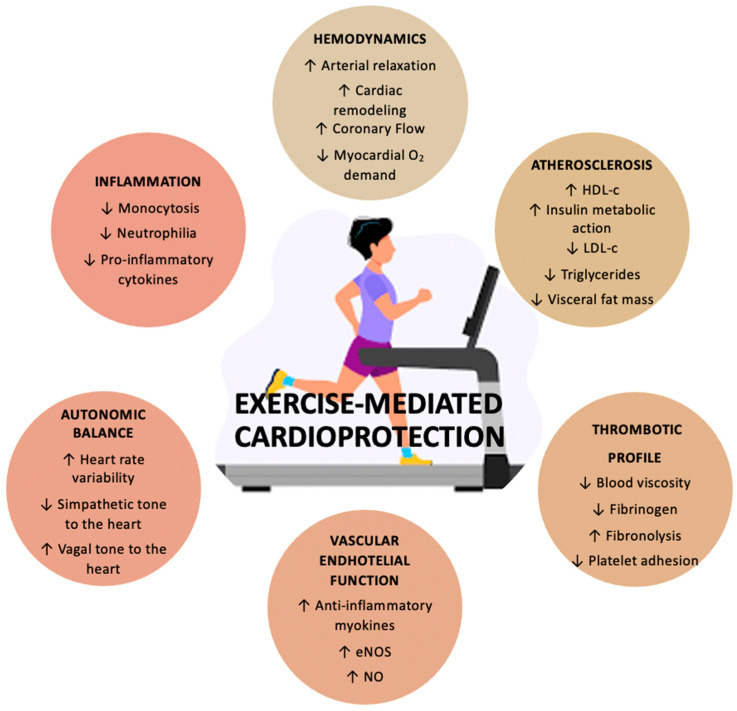
Exercise mediated protective effects on the cardiovascular system. Abbreviations: eNOS: endothelial nitric oxide synthase; NO: nitric oxide; HDL-c: high density lipoprotein cholesterol; LDL-c: low density lipoprotein cholesterol.

Specifically, physical activity promotes cardiovascular health through two main mechanisms: firstly by attenuating the negative effect of many established risk factors for cardiovascular disease (e.g., cholesterol, and insulin sensitivity) and secondly by exerting direct beneficial effects [18]. (Figure 1)

### 2.1. Effects on Cardiovascular Risk Factors

Regular training is associated with the reduction in cardiovascular risk factors, such as Body Mass Index (BMI), LDL cholesterol, blood pressure, and sleep apnea, and with the increase in HDL cholesterol and insulin sensitivity.

Exercise is known to be a powerful intervention for weight loss, particularly when combined with a balanced diet [19]. In turn, even a modest reduction in body weight (5–10%) ameliorates lipid disorders and other cardiovascular risk factors that generally coexist in dyslipidaemic patients [20]. Specifically, a meta-analysis that included 182 participants, using a random-effects model, showed a decrease in BMI in patients with sleep apnea receiving exercise prescriptions.

With regard to dyslipidemia, Kraus et al. explored the effects of the amount and the intensity of exercise on the lipid profile in overweight and obese dyslipidaemic patients, showing that high-amount–high-intensity exercise significantly reduced LDL concentrations. This effect was also confirmed for the HDL variables [21]; indeed, 25–30 km of brisk walking per week or the equivalent aerobic physical activities increased HDL levels by 3–6 mg/dL. Finally, in 509 T2DM patients, targeted metabolomics on lipidomics suggested that HDL subclasses appear sensitive to light intensities, whereas only the high category of physical activity intensity was consistently associated with VLDL subclasses [22].

With regard to blood pressure, a recent meta-analysis of data from 1207 hypertensive patients showed a significant reduction in systolic (−10 mmHg) and diastolic (−5.5 mmHg) blood pressure in subjects following aerobic training programs [23], with High-Intensity Interval Training (HIIT) also showing a significant reduction in older patients [24]; in addition, it has recently been shown that physical activity and exercise added to the usual care may further reduce BP in patients with resistant hypertension [25]; similar results have been observed in T2DM patients [26]. Similarly, strength training, by resulting in skeletal muscle hypertrophy, can lead to increased tissue responsiveness to insulin in terms of glucose storage and utilization. Thus, aerobic exercise, resistance training, or their combination can improve insulin sensitivity, leading to a reduced risk of developing T2DM in patients with impaired fasting glucose and reduced blood HbA1c concentrations (0.7%) in diabetic patients [27].

### 2.2. Direct Cardiovascular Effects

Regarding the direct effects of exercise on cardiovascular diseases (CVD), regular exercise exerts multiple positive effects on the structure and function of the heart and peripheral vasculature. Firstly, regular training is associated with higher nitric oxide bioavailability [28]. Ashor et al. reported the significant enhancement of endothelial function with aerobic, resistance, and combined modalities of exercises; for every increase of 2 metabolic equivalents (MET) in exercise intensity, a 1% improvement in flow-mediated dilation was observed [29]. Exercise also promotes vascular remodeling, which consists of increased diameter and dilatation capacity of the coronary and peripheral arteries and decreased vascular wall thickness and the development of coronary collateral vessels [30,31]. In addition, regular physical activity influences the atherosclerotic plaque structure by modifying the composition and amount of collagen and elastin in animal models [32]. Notably, other mechanisms by which exercise results in direct cardiovascular benefits involve the autonomic nervous system via a decrease in catecholamine levels, ß-adrenergic receptor concentration, angiotensin 2, and increased nitric oxide bioavailability, with a consequent protection against fatal arrhythmias. Life-threatening arrhythmias—rare in HFpEF—can also be prevented by cardiac preconditioning mechanisms [33]. Finally, among the direct cardioprotective mechanisms mediated by physical exercise, anti-thrombotic and anti-inflammatory properties play a crucial role and are mainly sustained by muscle-derived myokines [34].

### 2.3. Clinical Context

Exercise-related health benefits are reported in both primary and secondary prevention, including for patients with CAD or heart failure [35].

Numerous studies performed in primary-prevention patients have shown beneficial effects in CVD prevention regardless of age, gender, or ethnicity [36]. In a prospective cohort analysis among 4207 subjects, increased physical activity was inversely associated with coronary heart disease, stroke, and total cardiovascular disease, even in older patients. Risk reduction was related to the intensity and duration of exercise [37]. Manson et al. prospectively examined the incidence of cardiovascular events among postmenopausal women by physical activity, showing that walking and vigorous exercise were associated with a significant reduction in events, independently of ethnic group, age, and BMI [38]. The PRIME study collected data from 9758 patients and demonstrated that leisure-time physical activity energy expenditure was associated with a lower risk of major cardiovascular events [39]. Moreover, the ARIC study demonstrated that subjects who maintained guideline-recommended levels of physical activity over time had the lowest heart failure risk, and increasing physical activity over a 6-year interval was also associated with further risk reduction [40].

The positive effects of physical activity are also reported in secondary prevention, independently of age and disease severity. Cardiac rehabilitation (CR) reduces mortality, hospitalizations, and care use and improves cardiorespiratory fitness, quality of life, and mental health and is therefore strongly recommended in the international guidelines to patients with coronary artery disease [41,42,43,44,45]. The guidelines [35,46] suggest continuous aerobic exercise for at least 20–30 min 3 days/week (preferably 45–60 min 6–7 days/week) at 50–80% of VO_2_max. Resistance exercise two to three times/week should be added to the aerobic exercise. It consists of 8–10 exercises at an intensity of 30–70% of the 1 repetition maximum (1RM) for upper body exercises and 40–80% of 1RM for lower body exercises, with 12–15 repetitions in at least 1 set. The first meta-analyses of exercise-based CR was carried out over 30 years ago, demonstrating 20% to 25% reductions in CVD and all-cause mortality from ten randomized controlled trials in 4347 patients [47]. More recently, the meta-analysis by Lawler et al. of 34 randomized controlled trials summarized cardiovascular outcomes in post-MI patients who performed CR. The patients randomized to CR had a lower risk of reinfarction, cardiac mortality, and all-cause mortality, regardless of the study periods, the duration of CR, or the time beyond active intervention. In addition, CR had positive consequences on cardiovascular risk factors such as smoking habits, BMI, blood pressure, and lipid profile [48].

### 2.4. Effect of Exercise in Prevention of HFpEF

Physical activity prevents HFpEF through two main mechanisms: firstly by (i.e., indirect effects) attenuating the negative effect of many established risk factors for HFpEF (e.g., cholesterol, obesity, insulin sensitivity) and secondly (i.e., direct effects) by exerting beneficial effects on heart structure and cardiovascular performance (e.g., cardiac remodeling and cardiopulmonary performance) [49].

It has been well established that all the risk factors described above (see Section 2.1) predispose to HFpEF. Therefore, the ability of exercise to positively impact on these risk factors indirectly determines a decreased risk of HFpEF [49]. Specifically, a recent investigation investigating the association between exercise, body mass index, and HF in a cohort of more than 50,000 patients demonstrated that, among HF subtypes, the cumulative incidence of HFpEF was significantly lower across the higher physical activities categories; on the other hand, the association between higher levels of physical exercise and the cumulative risk of HFrEF was modest and not statistically significant [50]. A possible explanation for this can be found in the results of a study involving more than 20,000 subjects, showing that, after adjustment for HF risk factors (i.e., age, blood pressure, diabetes mellitus, body mass index, smoking status, and total cholesterol), a 1-unit greater fitness level in metabolic equivalents reached in midlife was associated with a lower risk of heart failure hospitalization after the age of 65, but with only a modest effect on the risk of coronary diseases [51]. These data further support the findings of a meta-analysis that revised twelve prospective cohort studies, including more than 370,000 subjects, demonstrating a dose-dependent inverse association between physical activity and the risk of HF [52].

However, low fitness and physical inactivity also negatively impact through direct effects on the HFpEF risk [49]; specifically, it has been shown that low fitness is associated with a higher prevalence of cardiac remodeling and diastolic dysfunction [53], suggesting that exercise may lower heart failure risk through its effect on favorable cardiac remodeling and the prevention of diastolic dysfunction; as a result, inactivity is correlated with diastolic dysfunction, determining a higher risk of HFpEF [49,53].

## 3. Evidence of Exercise in HFpEF

The HFpEF cardinal symptom is exercise intolerance (EI), manifested by dyspnea and fatigue during exertion, in some cases so limiting it forces patients into a sedentary lifestyle, further aggravating the clinical conditions [54,55]. Different systems contribute to EI in HF patients, influencing their treatment and prognosis [13]. Symptoms of EI and dyspnea were typically attributed to diastolic dysfunction, but multiple studies identified different cardiac abnormalities, including chronotropic incompetence and altered increase in systolic output in the context of increased left ventricular stiffness [56]. Moreover, recent findings highlighted the involvement of peripheral factors (such as vascular system, endothelium, adipose tissue, and skeletal muscle) in the pathophysiology of HFpEF, contributing to a significantly impaired ventricular–arterial coupling response to exercise [57,58,59,60,61].

In the following paragraphs, the clinical evidence available in HFpEF patients regarding the effect of exercise on different endpoints—evaluated with different techniques—will be discussed.

### 3.1. Cardiopulmonary Exercise Test

Historically, most studies evaluated exercise intolerance (primary endpoint to assess clinical status and the effects of therapeutic interventions in patients with HFpEF) through the cardiopulmonary exercise test (CPET); this method allows the contextual assessment of ventilatory, hemodynamic, and metabolic parameters [62,63,64]. Specifically, this technique quantifies EI by measuring the reduction in the peak oxygen consumption (VO_2_) (VO_2_ peak). The VO_2_ peak depends on peak cardiac output (Q peak) and the arteriovenous oxygen difference ((A-V) O_2_) peak, conforming to the Fick principle (VO_2_ peak = Q peak × A-V O_2_ peak) [62].

Several investigators identified peak VO_2_ as the primary outcome for assessing the effect of exercise in HFpEF (Table 1).. Kitzman DW et al. enrolled 53 elderly patients with HFpEF (EF ≥ 50% and no significant coronary, valvular, or pulmonary disease). In this study, the patients who performed supervised exercise training (3 days per week for 16 weeks) significantly increased VO_2_ peak exercise by 2.7 mL/Kg/min compared to the baseline, together with power output and exercise time compared to the control group (all *p* < 0.001) [65]. On the other hand, there were no changes in the peak respiratory exchange ratio between the groups. Similar findings were supported by the same groups in a later investigation, in which 32 HFpEF subjects performed endurance exercise training for 16 weeks [66]; as a result, the VO_2_ peak, the peak power output, and the ventilatory aerobic threshold (*p* = 0.01) were significantly higher in an active group than in the control group. A modest increase in the VO_2_ peak was observed by Fujimoto N in 11 HFpEF patients after 1 year of ET [67]. In the Prospective Aerobic Reconditioning Intervention Study (PARIS) study, in which 40 stable HFpEF patients were enrolled, an increase in peak VO_2_ of 3 mL/Kg/min was observed after 4 months of ET; specifically, an improvement of 16% was directly due to ET (as shown by differences in peak arterial–venous oxygen differences) [68]. An increase in the VO_2_ peak was also observed by Maldonado-Martin S in a population of 23 older patients performing cycling and walking at 50% to 70% of peak oxygen uptake intensity for 3 days/week for 16 weeks [69]. The VO_2_ peak was also considered as the primary endpoint in the multicenter trial performed by Edelmann F, in which 64 HFpEF patients were enrolled and 2:1 randomized to supervised endurance/resistance training, in addition to the usual care or to the usual care alone [70]. The exercise consisted of endurance by cycling 2 times weekly for 4 weeks and of resistance 3 times weekly from 5 inward training sessions (32 sessions). The authors found an increase in the VO_2_ peak of 2.6 mL/kg/min in the training group compared with a slight decrease of 0.7 mL/kg/min in the control group. Similar findings were reached by Brubaker PH, who demonstrated the enhancement of physical function in patients with HFpEF after 48 sessions of endurance exercise training, with an increase in the VO_2_ peak [71], and by Fu TC, investigating 30 HFpEF patients with aerobic interval training for 30 min/day, 3 days/week for 12 weeks on a cycle ergometer, reaching a significant post-interventional improvement of the VO_2_ peak (*p* < 0.05) [72]. Added to these data, the combination of caloric restriction and aerobic exercise training was related to exercise capacity, with a significant increase in the VO_2_ peak [73]. Finally, Smart NA determined the functional capacity responses of HFpEF patients to exercise training: in the exercise group, there was an incremental change in the VO_2_ peak (24.6%, *p* = 0.02) and a reduction in the V(E)/VCO_2_ slope (12.7%, *p* = 0.02) [74].

An additional field of interest is the investigation of the different effects of HIIT vs. moderate-intensity aerobic continuous training (MI-ACT) in HFpEF patients. Specifically, Angadi SS showed an increase in the VO_2_ peak by 9% after HIIT, from 19.2 ± 5.2 to 21.0 ± 5.2 mL/Kg/min, (*p* = 0.04), but it was unmodified after MI-ACT. The ventilation threshold, VE/VCO_2_ slope, peak HR, respiratory exchange ratio, VE/VCO_2_, and rate-pressure product were not changed in either group [75]. Similar findings were observed by Donelli da Silveira S and coworkers in a single-blinded randomized clinical trial; indeed, they found an improvement of VO_2_ of 22% in an HIIT group, compared with 11% with MI-ACT (*p* < 0.001) after a 12-week follow-up [76]. In contrast, a recent randomized controlled trial performed by Mueller et al. showed no differences in the VO_2_ peak between patients assigned to HIIT vs. MCT after 3 months [77].

In addition to peak VO_2_, other parameters (i.e., minute ventilation/carbon dioxide production (VE/VCO_2_) slop and ventilatory anaerobic threshold (VAT) have been investigate, as depicted in Table 2 and Table 3.

### 3.2. 6MWT

The effects of training programs on exercise tolerance have also been evaluated by performing the 6MWT in multiple RCTs (Table 4). The walking distance increased after exercise training [69,70,71,78], but no differences were observed when comparing the MCT or HIIT [75].

### 3.3. Echocardiography

Exercise-induced changes in diastolic function were widely investigated, with discordant evidence: on the one hand, some studies described no alterations in diastolic function, as assessed by the E/e’ medial, e’ medial, left atrial volume index; on the other hand, aerobic interval training reduced E/e’ E’ in HFpEF RCTs [67,68,72]. Alves et al. 2012 have shown in 31 HFpEF patients that exercise training 3 times/week for 6 months improves diastolic disfunction, with an increase in the E/A ratio and a decrease in the E-wave DT [79]. Similarly, Fu at al. reported a decrease in the E/e’ ratio with an enhancement of diastolic function after 12 weeks of aerobic interval training [72]. Moreover, the diastolic dysfunction grade was reduced after 4 weeks—HIIT (4 × 4 min at 85–90% peak heart rate, with a 3 min active recovery) in the study of Angadi [75], as well as in Donelli da Silveira, where the value of E/e’ decreased significantly in MCT and HIIT [76]. In contrast, Mueller analyzed diastolic function in HIIT, M-ACT, and a control group after 12 months and observed no significant differences among the groups [77]. Notably, no studies reported changes in the cardiac chamber dimensions. With regard to systolic function, in trials performed by Smart [74] and Kitzman [78] no significant changes were detected.

Table 5 depicts results of exercise training on echocardiographic parameters.

### 3.4. Circulating Biomarkers

In addition to diastolic dysfunction, the detection of elevated natriuretic peptide levels is crucial in the diagnosis of HFpEF [80,81]. No study reported a significant reduction in BNP or pro-BNP after programs of exercise training for HFpEF patients when compared to the controls. However, Donelli da Silveira et al. demonstrated a reduction in both groups in NT pro-BNP levels when HIIT and MCT were compared [76].

### 3.5. Quality of Life and Symptoms

Breathlessness and fatigue largely limit the quality of life of HFpEF patients. Exercise intolerance affects daily physical activities and impairs the mental and social quality of life. On this point, exercise training demonstrated the improvement of the quality of life, as assessed by using the Minnesota Living with Heart Failure Questionnaire (MLWHFQ) [66,70,71,72,78,82,83] and the Short Form Health Survey 36 (SF-36) [70,72,78,82], with an effect on the physical, emotional, and vitality domains [66,71].

According to the effect in QoL of different types of exercise training, Donelli da Silveira did not reveal significant differences of HIIT versus MI-ACT [76], while the recent trial by Mueller showed an improvement in the QoL domains in the MCT group compared with the controls, without significant differences between high-intensity interval training and the controls or high-intensity interval training and moderate continuous training [77].

### 3.6. Autonomic Dysfunction

HFpEF is also associated with autonomic dysfunction, manifesting as sympatho—vagal imbalance, which may result in alterations of heart rate variability (HRV). In this regard, Murad observed an improvement of the standard deviation of all normal RR intervals and of the root mean square of successive differences in normal RR intervals in older HFpEF patients performing 16 weeks of supervised training [84].

### 3.7. Brachial Artery Flow-Mediated Dilation (FMD)

Among the possible contributors to exercise intolerance in HFpEF patients, endothelial dysfunction and arterial stiffness seem to play a marginal role [67]- Table 6; even if it has been shown that HFpEF patients displayed typical arterial stiffness profiles (evaluated with the arterial velocity pulse index, AVI, and the arterial pressure volume index, API, at rest) when compared to HFrEF, with a significant negative correlation with peak VO_2_ [85], no changes in brachial artery flow-mediated dilation (FMD) were detected after exercise training in both the HIIT group and the MCT group [66,75]. These findings could be partially justified by the short training duration or the absence of endothelial damage. In addition, it is possible that the training exercise does not affect the vasodilatory function in HFpEF.

## 4. Clinical Impact and Future Perspectives

To date, the treatment of HFpEF is still a challenge for clinicians, and among the RCTs conducted on HFpEF patients, only a few have recently achieved the primary endpoints [86]. In this scenario, the scientific community focused on studying the role of physical activity in increasing exercise tolerance in HFpEF patients. Taken together, the analysis of these studies underlines two important findings: (1) exercise training programs improve the cardiorespiratory function of HFpEF patients, in terms of increase in peak oxygen uptake, the 6 min walk test distance, and the ventilatory threshold, as summarized in Table 1. As a result, it is essential to encourage adequate physical activity in these patients. (2) The diastolic or systolic functions are generally unchanged or only partially modified by the exercise, suggesting that multiple mechanisms contribute to the improvement of exercise tolerance in HFpEF patients. These mechanisms remain unclear, but some authors have suggested that the improvement in the VO_2_ peak after a structured exercise training in HFpEF patients is the expression of complex peripheral adaptation mechanisms and the consequent increase in oxygen extraction by skeletal muscle [66,87]. As a prototype, after 16 weeks of aerobic interval training, improvements in the VO_2_ peak were associated with a higher estimated peak arterial–venous oxygen difference and peak heart rate.

These findings reflect the multiple pathophysiological mechanisms determining the reduced exercise and functional capacity in HFpEF patients: cardiac impairment (chronotropic incompetence, reduction in left heart reserve capacity, and elevated filling pressures), vascular dysfunction, pulmonary impairment, and muscle diseases [13].

According to the literature, several studies explored different methods of training; notably, the studies are heterogenous for type, frequency, and intensity of training, including aerobic endurance exercise and resistance training, treadmill, walking, and bicycling. When compared, they did not show a clear superiority of one type over another. However, based on the data available in the literature, in stable HFpEF patients, the guidelines suggest initiating exercise training programs in supervised modalities [1,35,88]. The patients should perform continuous moderate-intensity endurance exercises, lasting from 20 to 60 minutes per session, 3 to 5 days per week, via cycling or treadmill modalities. The time and the frequency should be raised up before the intensity is increased. Once patients demonstrate tolerance for aerobic training levels, resistance training may be initiated.

Further investigations are needed to fully elucidate the pathophysiological mechanisms underpinning the shown beneficial effects of exercise in HFpEF. In addition, the proper role for different types of exercise is not yet understood, nor is the optimal time for exercise training in HFpEF. Moreover, there is a lack of data about long-term adherence and the combination of exercise with other behavioral and lifestyle interventions (e.g., nutrition). Finally, robust studies with a long follow-up are needed to demonstrate the role of exercise training in strong outcomes (i.e., mortality) in HFpEF patients.

Table 7 summarizes the clinical studies investigating the role of exercise training in HFpEF so far.

## 5. Conclusions

There is an intimate link between exercise and HFpEF, with the inability to perform physical activity being of the principal features impacting of the quality of life and outcome of HFpEF patients, in the presence of symptoms such as fatigue or dyspnea (i.e., exercise intolerance). Considering that exercise training programs are able to improve the cardiorespiratory function of HFpEF patients through multiple mechanisms, even if no role has demonstrated an amelioration in the prognosis, the prescription of exercise training programs in supervised modalities, to improve symptoms, quality of life, and exercise tolerance, should be recommended for stable HFpEF patients.

## Figures and Tables

**Table 1:** Results of trials investigating the effects on peak VO_2_ of exercise training in HFpEF.

Peak VO_2_ mL/kg/min	ET		Ctr		
	**Baseline**	**Final**	**baseline**	**Final**	***p* ^§^**
*Kitzman 2010* [78]	13.8 ± 2.5	16.1 ± 2.6 *	12.8 ± 2.6	12.5 ± 3.4	<0.001
*Edelmann 2011* [70]	16.1 ± 4.9	18.7 ± 5.4 *	16.7 ± 4.7	16.0 ± 6.0	<0.001
*Smart 2012* [74]	12.2 ± 3.6	15 ± 4.9 *	14.1 ± 4.1	14.8 ± 4.6	0.06
*Kitzman 2013* [66]	14.2 ± 2.8	15.8 ± 3.3	14.0 ± 3.2	13.8 ± 3.1	0.0001
*Maldonado-Martin 2017* [69]	13.5 ± 2.3	16.0 ± 2.6 *	12.7 ± 3.2	12.6 ± 3.4	0.01
*Brubaker 2020* [71]	13.7 ± 2.8	15.2±6.3 *	13.3 ± 3.0	13.0–14.0	0.001
	**HIIT**		**MCT**		
	**Baseline**	**final**	**baseline**	**Final**	***p* ^§^**
*Angadi 2015* [75]	19.2 ± 5.2	21.0 ± 5.2 *	16.9 ± 3.0	16.8 ± 4.0	d: 0.94 vs. −1.63
*Donelli da Silveira 2020* [76]	16.1 ± 3.3	19.6 ± 3.5 *	17.6 ± 3.5	19.5 ± 3.7 *	<0.001
*Mueller 2021* [77]	18.9 ± 5.4	20.2 ± 6.0	18.2 ± 5.1	19.8 ± 2.5	0.002

ET: exercise training; Ctr: control group; HIIT: high-intensity interval training; MCT: moderate continuous training. * *p* < 0.05 pre-intervention vs. post-intervention in the same group; ^§^ *p*: post-intervention between the groups.

**Table 2:** Results of trials investigating the effects on minute ventilation/carbon dioxide production (VE/VCO_2_) slope of exercise training in HFpEF.

*VE/VCO_2_ Slope*	ET		Ctr		
	**Baseline**	**final**	**baseline**	**Final**	***p* ^§^**
*Kitzman 2010* [78]	34 ± 6	35 ± 8	33 ± 5	34 ± 5	n.s.
*Smart 2012* [74]	33.9 ± 3.3	29.6 ± 5.3	33.7 ± 3.0	33.8 ± 3.2	n.s.
*Kitzman 2013* [66]	31.5 ± 4.4	32.2 ± 4.5	30.6 ± 3.6	30.2 ± 3.3	n.s.
	**HIIT**		**MCT**		
	**Baseline**	**final**	**baseline**	**final**	***p* ^§^**
*Angadi 2015* [75]	31.2 ± 11.5	31.6 ± 10.3	26.5 ± 2.4	26.7 ± 3.1	n.s.
*Donelli da Silveira2020* [76]	39.4 ± 6.1	35.7 ± 4.7	36.8 ± 5.4	34.6 ± 5.1	<0.001
*Mueller 2021* [77]	34.5 ± 7.9	35.0 ± 9.8	34.2 ± 7.2	33.7 ± 6.8	n.s.

ET: exercise training; Ctr: control group; HIIT: high-intensity interval training; MCT: moderate continuous training. ^§^ *p*: post-intervention between the groups.

**Table 3:** Results of trials investigating the effects on ventilatory anaerobic threshold (VAT) of exercise training in HFpEF.

*Ventilatory Anaerobic Threshold (VAT)*	ET		Ctr		
	**Baseline**	**final**	**baseline**	**final**	***p* ^§^**
*Kitzman 2010* [78] (mL/min)	746 ± 149	822 ± 180 *	660 ± 174	618 ± 126	<0.001
*Eldemann 2011* [70] (mL/min/kg)	10.2 ± 3.0	12.7 ± 3.6 *	10.3 ± 2.5	10.0 ± 3.2	<0.001
*Smart 2012* [74] (mL/min/kg)	7.8 ± 1.8	9.8 ± 2.6	9.1 ± 3.8	9.2 ± 5.3	n.s.
*Kitzman 2013* [66] (mL/min)	699 ± 178	796 ± 163 *	734 ± 189	702 ± 186	0.01
*Maldonado Martin 2017* [69] (mL/min/kg)	9.3 ± 1.5	10.4 ± 1.4	8.3 ± 1.3	8.3 ± 2.2	n.s.
*Brubaker 2020* [71] (mL/min)	721 ± 161	768.8 ± 846.2 *	703 ± 184	636.5 ± 711.5	0.001
	**HIIT**		**MCT**		
	**Baseline**	**final**	**baseline**	**final**	***p***
*Angadi 2015* [75] ml/min/kg	12.2 ± 4.0	13.1 ± 3.5	11.1 ± 2.1	11.7 ± 2.4	n.s.

ET: exercise training; Ctr: control group; HIIT: high-intensity interval training; MCT: moderate continuous training. * *p* < 0.05 pre-intervention vs. post-intervention in the same group; ^§^ *p*: post-intervention between the groups.

**Table 4:** Results of trials investigating the effects on 6-minute walking distance of exercise training in HFpEF.

6MWT Distance (m)	ET		Ctr		
	**baseline**	**final**	**baseline**	**final**	***p* ^§^**
*Kitzman 2010* [78]	1494 ± 224	1659 ± 173 *	1412 ± 382	1460 ± 411	0.002
*Edelmann 2011* [70]	545 ± 86	569 ± 88 *	551 ± 86	568 ± 80	0.63
*Kitzman 2013* [66]	447 ± 107	486 ± 89	438 ± 79	448 ± 70	0.009
*Maldonado-Martin 2017* [69]	455 ± 68	506 ± 53 *	402 ± 142	430 ± 125 *	0.028
*Brubaker 2020* [71]	445 ± 88	474.5–504.1 *	425 ± 117	434.4 ± 462.9	<0.001

* *p* < 0.05 pre-intervention vs. post-intervention in the same group; ^§^ *p*: post-intervention between the groups. ET: exercise training; Ctr: control group.

**Table 5:** Results of trials investigating the effects on echocardiographic parameters of exercise training in HFpEF.

ECHOCARDIOGRAPHY	ET		Ctr		
	**Baseline**	**final**	**baseline**	**final**	***p* ^§^**
*Kitzman 2010* [78]					
EF (%)	61 ± 5	57 ± 8	60 ± 10	55 ± 8	ns
E/A	0.90 ± 0.24	1.02 ± 0.28	1.02 ± 0.38	1.12 ± 0.36	ns
DT	220 ± 55	230 ± 40	227 ± 52	221 ± 52	ns
*Edelmann 2011* [70]					
EF (%)	67 ± 7	66 ± 6	66 ± 7	67 ± 8	ns
E/e’	12.8 ± 3.2	10.5 ± 2.5 *	13.5 ± 4.6	14.1 ± 3.9	0.01
LAVi mL/m^2^	27.9 ± 7.6	24.3 ± 6.5 *	28.2 ± 8.8	28.6 ± 9.2	0.01
*Smart 2012* [74]					
EF (%)	58 ± 13.2	61.3 ± 9.5	56.7 ±7.7	58.7 ±6.4	ns
E/A	0.87 ± 0.13	0.82 ± 0.17	0.94 ± 0.39	0.82 ± 0.22	ns
DT	276 ± 50	281 ± 54	245 ± 44	248 ± 36	ns
E/e’	20.7 ± 12.8	25.1 ± 24	15.9 ± 6.8	15.9 ± 5.5	ns
*Alves 2012* [79]					
EF (%)	56.4	57.7 *	55.9	55.4	0.01
E/A	0.93	1.05 *	1.01	1.04	0.01
DT (sec)	236.7	222.7 *	216.9	214.8	0.01
EDd (mm)	51.5	51	51.7	51.9	ns
ESd (mm)	30.4	29.6	30.9	31.3	ns
*Fu 2016* [72]					
EF (%)	57.6 ± 1.9	57.8 ± 1.7	56.5 ± 2.2	54.4 ± 3.3	ns
E/A	1.1 ± 0.2	0.9 ± 0.1	1.0 ± 0.3	0.8 ± 0.2	ns
E/e’	21.0 ± 2.2	16.1 ± 1.8*	19.2 ±1.5	17.8 ± 1.9	ns
	**HIIT**		**MCT**		
*Angadi 2015* [75]					
EF (%)	65 ± 5	63 ± 6	66 ± 4	61 ± 5	ns
E/A	1.3 ± 0.5	1.2 ± 0.5	1.2 ± 0.6	1.6 ± 1.1	ns
*DT*	194 ± 55	225 ± 40 *	199 ± 71	220 ± 43	ns
E/e’	14.6 ± 5.6	12.7 ± 4.7	17.7 ± 6.3	16.7 ± 5.2	ns
*LAVi* (mL/m^2^)	35.8 ± 3.0	32.4 ± 7.2	40.5 ± 9.3	46.3 ± 18.1	ns
*Donelli da Silveira 2020* [79]					
EF (%)	65 ± 5	66 ± 4	65 ± 5	65 ± 5	ns
E/A	0.99 ± 0.2	0.91 0.2	1.05 ± 0.3	1.08 ± 0.3	ns
*DT*	233 ± 33	222 ± 27	214 ± 33	209 ± 37	ns
E/e’	14.2 ± 4	11.6 ± 3 *	13.3 ± 3	11.1 ± 2 *	ns
*Mueller 2021* [77]					
E/e’	15.8 ± 3.7	14.2 ± 3.9	15.9 ± 4.1	15.6 ± 4.4	ns
LAVi mL/m^2^	35.4 ± 9.0	37.4 ± 10.9	37.9 ± 13.0	36.6 ± 9.2	ns

ET: exercise training; Ctr: control group; EF: ejection fraction; E: mitral peak early wave velocity; A: mitral peak atrial/late wave velocity; LAVi: left atrial volume indexed; DT: deceleration time; e’: early diastolic mitral annular tissue velocity; EDd: end-diastolic diameter; ESd: end-systolic diameter. * *p* < 0.05 pre-intervention vs. post-intervention in the same group; ^§^ *p*: post-intervention between the groups.

**Table 6:** Results of trials investigating the effects on brachial artery flow-mediated dilation of exercise training in HFpEF.

Brachial ArteryFlow-Mediated Dilation (%)	ET		Ctr		
	**baseline**	**final**	**baseline**	**Final**	***p* ^§^**
*Kitzman 2013* [66]	4.0 ± 2.0	3.8 ± 3.0	4.7 ± 3.5	4.3 ± 3.5	ns
	**HIIT**		**MCT**		
	**baseline**	**final**	**baseline**	**Final**	
*Angadi 2015* [75]	6.9 ± 3.7	7.0 ± 4.2	8.1 ± 4.1	3.4 ± 3.6	ns

^§^*p*: post-intervention between the groups; ET: exercise training; Ctr: control group.

**Table 7:** List of studies investigating the role of exercise training in patients with preserved ejection fraction.

*First Author; Year of Publication (Ref.)*	Study Design; Duration (wk)	Type of Exercise	Patient; Age; Women (%)	Principal Outcomes Investigated and Main Findings
*Kitzman2010* [78]	Randomized, prospective, attention-controlled, single-blind study 16 wk	1 h ET (warm-up, stimulus, and cool-down phases), 3 times/wk	ET UC	CPET functional parameters 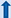 Echocardiographic parameters 6MWT distance 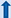 Natriuretic peptides QoL scores 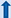
			N = 26 N = 27	
			70 ± 6 y 69 ± 5 y	
			83% 91%	
*Edelmann2011* [70]	Prospective, randomized 2:1 controlled trial 12 wk	Endurance/resistance training	ET UC	CPET functional parameters 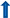 Echocardiographic parameters 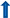 6MWT distance QoL scores
			N = 44 N = 20	
			64± 8 y 65 ± 6 y	
			55% 60%	
*Smart2012* [74]	Randomized, controlled trial 16 wk	Cycle ergometer exercise training at 60 rpm	ET UC	CPET functional parameters 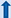 Echocardiographic parameters 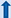 QoL scores
			N = 12 N = 13	
			67 ± 5.8 y 61.9 ± 6.9 y	
			42% 53%	
*Murad2012* [84]	Randomized, controlled, single-blinded design 16 wk	1 h ET (warm-up, stimulus, and cool-down phases), 3 times/wk	ET UC	SDNN 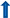 RMSSD 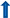
			N = 35 N = 31	
			70.1 ± 5.6 y 68.0 ± 4.8 y	
			63% 64.5%	
*Alves2012* [79]	Randomized controlled trial 24 wk	Interval training	ET UC	Exercise tolerance (MET) 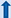 Echocardiographic parameters 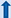
			N = 20 N = 11	
			Overall	
			63 ± 11 y	
			29%	
*Haykowsky2012* [68]	Randomized, single-blind trial 16 wk	Endurance exercise training	ET UC	CPET functional parameters 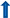 Echocardiographic parameters
			N = 22 N = 18	
			70 ± 6 y 68 ± 5 y	
			82 % 94%	
*Fujimoto2012* [67]	Randomized controlled trial 54 wk	Endurance exercise training	ET UC	CPET functional parameters Echocardiographic parameters Cardiac catheterization measurements
			N = 7 N = 13	
			74.9 ± 6 y 70.2 ± 4 y	
			57% 38%	
*Kitzman2013* [66]	Randomized, controlled, single-blind trial 16 wk	Walking, arm, and leg ergometry	ET UC	CPET functional parameters Echocardiographic parameters 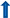 Brachial artery FMD QoL scores
			N = 32 N = 31	
			70 ± 7 y 70 ± 7 y	
			72% 80%	
*Angadi2015* [75]	Randomized comparison trial/pilot study 4 wk	HIIT vs. MI-ACT	HIIT MCT	CPET functional parameters 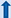 Diastolic function 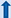 FMD
			N = 9 N = 6	
			69.0 ± 6.1 y 71.5 ± 11.7 y	
			11% 33%	
*Nolte2015* [83]	Prospective, randomized, controlled trial 12 wk	Endurance/resistance training	ET UC	QoL scores 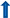
			N = 44 N = 20	
			overall	
			65 ± 7 y	
			56%	
*Kitzman2016* [73]	Randomized, attention- controlled, 2 × 2 factorial trail 20 wk	Walking exercise 3 times/wk	ET Diet group	CPET functional parameters 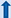 QoL scores
			N = 26 N = 24	
			ET + Diet group UC	
			N = 25 N = 25	
			Overall	
			67 ± 5 y	
			81%	
*Fu2016* [72]	Randomized, controlled trial 12 wk	Aerobic interval training	ET UC	CPET functional parameters 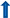 Echocardiographic parameters QoL scores 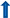
			N = 30 N = 30	
			60.5 ± 2.7 y 63.1 ± 2.6 y	
			33% 40%	
*Maldonado-Martin2017* [69]	Prospective, randomized, single-blinded trial 16 wk	Cycling and walking at 50% to 70% of VO_2_ peak intensity	ET UC	CPET functional parameters 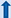 6MWT distance
			N = 23 N = 24	
			>65 y	
			87%	
*Lang2018* [83]	Randomized controlled trial 12 wk	Home-based comprehensive self-management rehabilitation program	ET UC	QoL scores 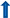
			N = 25 N = 25	
			71.8 ± 9.9 y 76 ± 6.6 y	
			64% 44%	
*Donelli da Silveira2020* [76]	Single-blinded, parallel randomized clinical trial 3 d per wk 12 wk	HIIT vs. MCT	HIIT MCT	CPET functional parameters 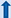 Echocardiographic data Natriuretic peptides 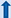 QoL scores 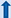
			N = 10 N = 9	
			60 ± 10 y 60 ± 9 y	
			70% 56%	
*Brubaker2020* [71]	Randomized controlled trial 16 wk	≤60 min of moderate-intensity endurance exercise training 3 time/wk	ET UC	CPET functional parameters 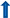 6MWT distance 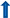 QoL scores
			N = 58 N = 58	
			70.3 ± 6.7 69.2 ± 6.2	
			76% 86%	
*Mueller2021* [77]	Randomized controlled trial 3 mo supervised followed by 9 mo of telemedical monitored home-based training	HIIT vs. MCT vs. UC	HIIT MCT UC	CPET functional parameters 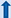 Diastolic function Natriuretic peptides QoL scores
			N = 58 N = 58 N = 60	
			70 ± 7 y 70 ± 8 y 69 ±10 y	
			71% 60% 68%	

*Abbreviations*: ET, exercise training; wk, weeks; CPET: cardiopulmonary exercise test; 6MWT: 6 min walking test; FMD, flow-mediated dilation; HIIT, high-intensity interval training; IMT, inspiratory muscle training; MET, metabolic equivalent task; MCT: moderate continuous training; MI-ACT, moderate-intensity aerobic continuous training; QoL, quality of life; RMSSD, root mean square of successive differences in normal RR intervals; SDNN, standard deviation of all normal RR intervals; UC, usual care. The blue arrows indicate an improvement after exercise of the variable explored

## Data Availability

Not applicable.
